# Influence of kaolin application on most important fruit and leaf characteristics of two apple cultivars under sustained deficit irrigation

**DOI:** 10.1186/s40659-020-00325-z

**Published:** 2021-01-06

**Authors:** Somayeh Faghih, Zabihollah Zamani, Reza Fatahi, Mohammad Omidi

**Affiliations:** Department of Horticulture Science, College of Agriculture and Natural Resources, University of Tehran, Karaj, 31587 Iran

**Keywords:** Drought stress, Malondialdehyde, Proline, Fruit weight, Fruit firmness, Soluble solids

## Abstract

**Background:**

Apple is one of the oldest and most valuable fruits. Water restriction is one of the major problems in the production of this fruit in some planting areas.

**Methods:**

Effects of kaolin spray treatments were studied on two early apple cultivars of Golab and Shafi-Abadi under sustained deficit irrigation (SDI) in Alborz province, Iran during 2017 and 2018. Irrigation treatments were 100%, 85%, and 70% ETc and kaolin application were concentrations of 0, 3 and 6% in 2017 and 0, 1.5 and 3% in 2018.

**Results:**

Results showed that 85% ETc treatment compared to other irrigation treatments improved apple tree crown volume in 2017. Deficit irrigation treatments significantly reduced fruit weight in both years. Application with 6% kaolin resulted in 33.3% increase in apple fruit weight compared to non-kaolin treatment at 100% ETc irrigation in the first year. Severe deficit irrigation (70% ETc) significantly reduced apple fruit length in both years, but 6% kaolin increased fruit length in both apple cultivars in 2017. Severe deficit irrigation treatment increased the firmness of apple fruit compared to control and mild deficit irrigation (85% ETc) in the first year of experiment. There was no significant difference between irrigation treatments for apple fruit firmness in the second year of experiment. Kaolin treatments of 1.5% and 3% at full irrigation increased the soluble solids content of apple fruit by 36.6% and 44.1% in 2018, respectively. Deficit irrigation treatments significantly increased leaf proline content compared to control in both years. In the first year, kaolin treatments increased leaf proline but in the second year, leaf proline was not significant. Deficit irrigation treatment of 70% ETc and 6% kaolin had the highest amount of glycine betaine content, malondialdehyde and hydrogen peroxide in apple leaf in the first year of experiment.

**Conclusions:**

Severe deficit irrigation stress (70% ETc) increased the activity of nonenzymatic defense systems of apple trees. Kaolin as a drought stress reducing agent can be recommended in apple orchards of Golab and Shafi-Abadi cultivars as an effective and inexpensive method to improve tolerance to drought stress conditions.

## Background

Apple (*Malus domestica* Borkh) is the most important fruit of temperate regions, due to its delicious taste and low-calorie with significant amounts of beneficial compounds. In order to sustain the world growing population, agricultural production needs to be increased, however the fresh water needed for agriculture is declining. Therefore, efficient strategies to increase crop water productivity in arid and semi-arid regions are of great importance. In recent years, deficit irrigation (DI)) methods or irrigation less than normal have been studied as valuable methods for arid regions reduce the water used for crop cultivation [1]. In the sustained deficit irrigation (SDI) method, irrigation is applied in less than normal uniformly throughout the growing season. In fact, in SDI, water shortage stress on plant occurs at all stages of development [2]. Although light sandy loam soils are well suited for fruit orchards, nutrients and pesticide chemicals leaching into groundwater is more common in these soils. Therefore, both nutrients and chemicals are removed from the roots zone and the risk of environmental pollution increases. Hence, DI reduces fertilizer and pesticides use and prevents groundwater contamination [3]. Vegetative growth in woody plants is known to be the most sensitive process to DI. Decrease in branch length growth responses to DI that lead to reduction in tree size [4]. As vegetative growth declines, light penetration into the tree canopy can be increased, allowing the fruit to color better [5]. Increasing the penetration of light into the canopy of peach trees increases the color of its fruit. In fruits, the formation of yellow color depends on the breakdown of chlorophylls and production of carotenoids and the red color is mostly due to anthocyanins [6]. Chlorophylls break down to form yellow color and development of red color stops in the presence of high nitrogen. With DI, nitrogen content in apple and pear fruit decreased, so helping in the formation of appropriate fruit color [7]. Severe water deficit stress decreased vegetative growth of the tree which is due to increased growth inhibitory hormones and reduced growth stimulating hormones, meanwhile reduced fruit quality and the attack of plant pests [8, 9]. In the event of water stress, fruit growth decreases due to restricted cell division and cell size, while fruit firmness may be increased [10]. Studies also show that due to the occurrence of water deficit stress the amount of soluble solids increases in fruits. DI stress accelerates fruit starch degradation and increases ethylene production in fruit [7, 11]. Fruit tissue firmness is an important attribute of apple that is affected by fruit maturity and size. With the ripening progress of apple and pear fruits, their firmness decreases. Smaller fruits tend to be firmer than larger fruits due to their higher cell density. Treatments such as DI that reduce fruit size increase the fruit firmness. The content of soluble solids and acids have a significant effect on apple fruit flavor. Significant increases in soluble solids of apple, peach and pear fruits have been observed under drought stress conditions [7, 12]. In a report regulated deficit irrigation (RDI) as 50% of complete irrigation at the last stage of apple fruit growth (whit full irrigation before that) was compared with SDI during a three year period. RDI had no effect on yield loss and fruit size, but SDI throughout the growing season reduced fruit size [13]. In peach tree, it has been shown that while water content in fruit is highly susceptible to DI at all stages of fruit growth, dry matter is relatively insensitive to it. It has also been shown that severe irrigation restriction at the late stages of peach fruit growth reduces fruit size, resulting in reduced yield [14]. In grape, proper and full irrigation, equal to the rate of evapotranspiration, may increase the number and size of berries, but at the same time will reduce fruit quality. Impaired irrigation after the onset of grape ripening showed little effect on fruit size but improved fruit compounds such as anthocyanins [15–17].

Proline is one of the non-enzymatic antioxidants commonly increased during drought stress having role in eliminating ROS, protecting the cell membranes and antioxidant enzymes [9]. Proline as a compatible osmolyte can accumulate in large quantities without damage to cellular macromolecules and used as a source of carbon and nitrogen in the cell [18]. Under drought stress in olive, young leaves had higher photosynthetic rates while older leaves had high levels of proline and soluble sugars, suggesting that older leaves had a protective role for water retention in plant and hence the continue of carbon assimilation in younger leaves [18].

Another non-enzymatic antioxidant in the plants to counter environmental stresses is glycine betaine. This compound increases stress tolerance by preventing lipid degradation and maintaining osmotic balance. Under water deficit conditions, glycine betaine can protect photosynthetic systems including photosynthetic enzymes, proteins and lipids in thylakoid membranes and electron flow in photosystem II.

Kaolin is a natural neutral clay not toxic to plants and animals and its spray application provides a uniform thin layer coating protector on the plant. The coating created by this material is porous, does not prevent gas exchange and the closure of leaf apertures, permits the photosynthetic active radiation, but partially prevents infrared and ultraviolet radiation pass to plant. Kaolin is easily washed from crops and is also used to protect plants against pests and diseases by changing the behavior of insects and pathogens [19]. Kaolin reduces the temperature of the canopy about two to six Celsius degrees by reflecting part of the light reaching the canopy. This decrease in temperature will reduce the harmful effects of drought stress, and can increase photosynthesis, carbohydrate transfer to the fruit, and fruit anthocyanins [20, 21]. In apple trees under DI, with decreasing irrigation levels vegetative growth traits including shoot length and stem diameter decreased but kaolin application improved these traits [22]. Application of kaolin, increased sugar and anthocyanin accumulation in fruit and improved red color of apple. Researchers believe that the main effect of temperature on anthocyanin formation is its effects on the activity of PAL (Phenylalanine ammonia-lyase) enzyme, in such a way that high temperature decreases the activity of this enzyme but low temperature increases its activity [23, 24]. Kaolin particle film application in the Mediterranean region had a positive effect on increasing olive tree yield resulted by the increase in the weight and size of the available fruit [25]. Kaolin by reflecting intense light and decreasing plant surface temperature increased also yield in apples [26, 27], grapefruit [28], pomegranates [29] and walnuts [30]. In apple trees sprayed with kaolin, incidence of powdery mildew decreased, fruit had a higher firmness and calcium content while decreased storage disorders such as bitter pit and water core [31].

Due to the scarcity of water resources for agricultural and horticultural purposes in dry areas, irrigation planning with the aim of determining proper irrigation to save water resources while improve the yield and quality and increase water productivity is essential. Also, the combination of kaolin particle film application with irrigation methods is of particular importance in hot areas. Hence, this study was conducted in accordance with this aim on two apple cultivars.

## Materials and methods

### Experiments site and plant material

present study was conducted over two consecutive years of 2017 and 2018 on apple trees ‘Golab’ and ‘Shafi-Abadi’ grafted on seedling rootstocks, with 25 years of age and 6 m × 4 m planting distance in Karaj area of Alborz Province (35° 48′ N, 50° 57′ E, 1293 M elevation). These two apple cultivars are of the most important Iranian apple cultivars due to their early ripening and special taste, which are marketed in late spring and early summer. Average year temperature, total precipitation and evaporation in 2017 were 16.4 °C, 169.3 mm and 1431.8 mm, respectively. Soil characteristics of the experiment site is reported previously in Faghih et al. [22].

### Irrigation and kaolin treatments

The irrigation system was drip, with seven emitters for each tree, and each with a flow rate of 4 L per hour. The irrigation interval for all treatments was 3 days. Experimental treatments included irrigation to the full water requirement (100% crop evapotranspiration, ETc), and sustained deficit irrigation at two levels of 85% and 70% ETc during the growing season (from a month after full bloom to October).

Moisture was measured in the root development depth by installing a TDR instrument (Time Domain Reflectometer, Mini Trase, California (USA)).

The moisture meter readings were performed before and after each irrigation. Water requirement was determined by determining soil moisture before irrigation using I = ETc = (θfc−θi) DR equation [32]. In this equation, I is irrigation water depth (mm), θfc water content at field capacity, θi soil moisture before irrigation, and DR is depth of apple tree root development.

The kaolin used in this experiment was processed kaolin (Sepidan WP, Kimia Green Company, Iran) as a water suspension at three levels of 0, 3 and 6% concentrations in 2017 and 0, 1.5 and 3% in 2018. Spraying was performed as full canopy cover for 3 times (May 23, 2 weeks and 2 months later).

### Sampling and measuring the evaluated traits

#### Measuring tree crown volume

Crown volume of the trees was calculated at the end of the growing season before leaf fall (early October 2017) by measuring the diameter and height of the crown using the following equation [33].

$${\text{TCV = 4/3}}\pi {\text{ab}}^{ 2}$$

π: 3/14, a: 1/2 large axis and b: 1/2 small axis.

#### Physical properties of fruits

By random selection of 15 healthy and uninjured fruits in each treatment (5 fruits per replicate), the weight of each was accurately measured by digital scale (BP2IID model, Germany) and the height and diameter of the fruit by digital caliper (Model DT209, Japan). Firmness of the fruit tissue after removing the skin of the fruit was measured from two opposite sides of the middle part using a Penetrometer (Stable Microsystems Texture Technologies Inc., UK) with an eight mm head and expressed in kg/cm^2^. A colorimeter (Konica Minolta CR-403, Japan) was used to measure color parameters a*, b* and L*. The number of fruits infected with apple moth in each tree (based on the visible appearance) was counted accurately after harvest, and presented as percentage of all fruits.

#### Chemical properties of fruits

At harvest, the chemical properties of 15 healthy fruits were measured. The moisture content of the fruit was calculated based on the difference of fresh weight of the fruit with its dry weight after 3 days drying in the oven at 70 °C [34]. The soluble solids content was measured by a manual refractometer (Atago, Japan) at room temperature as the percentage of brix. To measure the titrable acids, 10 ml of the filtered fruit juice of each replicate of each treatment was prepared and diluted with distilled water to 50 ml, then titrated with 0.1 N NaOH until the pH of 8.2 and the amount of NaOH was used for calculating the acids content as malic acid using the following equation [35].

$${\text{A = }}\frac{{{\text{S}}\; \times \;{\text{N}}\; \times \;{\text{E}}\; \times \;{\text{F}}}}{\text{C}} \times 1 0 0$$

A equals to organic acids content as malic acid in the fruit extract (g/100 ml), S the volume of NaOH [36], N normality of NaOH (0.1 normal), F the normal coefficient equal to one, C the fruit extract used for titration (10 ml) and E is the normal weight of the acid (0.67 for malic acid). Fruit flavor index calculated as total soluble solids (TSS)/total acids (TA) [35].

#### Leaf relative water content

Samples of apple fresh leaves were weighted with a digital balance then were immersed in distilled water at 4 °C for 24 h for full imbibition. Leaves were then removed and after absoption of surface water they were weighed again. Leaf samples were then dried in an oven at 75 °C for 48 h and their dry weight was measured. Relative water content was calculated using the following equation [37].

$${\text{RWC}} = 100 \times \left( {{\text{W}}_{\text{F}} - {\text{W}}_{\text{D}} } \right)/\left( {{\text{W}}_{\text{T}} - {\text{W}}_{\text{D}} } \right)$$

In the above equation: RWC is relative water content, W_F_ leaf fresh weight, W_D_ leaf dry weight, W_T_ leaf turgor weight.

#### Leaf proline and glycine betaine

To measure proline, half a gram of grinded fresh leaf sample was added into a falcon tube, then 5 ml of 3% sulfosalicylic acid was added, and the tubes were placed on ice. The falcons were centrifuged at 9000*g* for 4 min at 4 °C. Then 1 ml of the supernatant transferred into new falcon and 1 ml ninhydrin acid solution (1.25 g ninhydrin, 30 ml glacial acetic acid and 20 ml 6 mM phosphoric acid) and 1 ml glacial acetic acid were added and vortexed. Then, 2 ml of toluene was added to the solution and the absorbance was measured at 520 nm. By using a standard curve and its line equation, the amount of proline in the samples were determined in micrograms/ml. The standard curve (correlation coefficient of R^2^ = 0.992) was prepared using pure l-proline at concentrations of 0, 8, 16, 24, 32, 40 and 48 μmol/ml and pure toluene was used as blank. Using the following equation, proline content was expressed in micromoles per gram of fresh leaf weight [38].

$${\text{Proline content }}({\text{as }}\mu {\text{mol}}/{\text{g Fresh weight}} = \, [\mu {\text{g Proline}}/{\text{ml}}/ 1 1 5. 5 { }\mu {\text{g}}\;\mu {\text{mol}}^{ - 1} \left] / \right[{\text{g Samples}}/5]$$

In the above formula, Proline content: proline content in micromol per gram fresh leaf tissue, μg Proline/ml: Proline value obtained using standard curve, µg µmol^−1^: Proline molar mass and: g Samples the weight of the plant sample (0.5 g).

To measure glycine betaine (GB), half a gram of fresh leaf was powdered and added into the falcon and was shaken with 20 ml of deionized water for 48 h at 25 °C. Samples were filtered through a filter paper and stored in − 20 °C until measurement. For measurement, the samples were diluted at 1: 1 ratio with 2 normal sulfuric acid (H_2_SO_4_), (250 μl of extract with 250 μl of sulfuric acid) and the tubes were cooled on ice for 60 min. Then 0.2 ml of potassium iodide (KI-I_2_) was added to the solutions. The samples were then placed in the refrigerator for 24 h. After this period, samples were centrifuged at 10,000*g* for 15 min at 0 °C. The supernatants were then discarded and periodide crystals obtained were dissolved in 9 ml of dichloro-ethane. After the solutions were kept at room temperature for 2–2.5 h, their absorbance at 365 nm was measured by a plate reader (EON, Bio Tek America) and the content of glycine betaine determined against standard curve and the equation of glycine betaine concentrations (7 to 100 μg/ml). Using the following equation, the amount of glycine betaine was expressed in micromoles per gram of fresh weight [39].

$${\text{GB content }}\left( {\mu {\text{mol g}}^{ - 1} {\text{F}}.{\text{ W}}.} \right) = \, \left[ {\left( {\mu {\text{g GB ml}}^{ - 1} *{\text{ ml Dichloro}} - {\text{ethane}}} \right)/ 10 2 { }\mu {\text{g }}\mu {\text{mol}}^{ - 1} } \right]/\left[ {\left( {\text{g sample}} \right)} \right]$$

In the above formula, GB content: Tissue glycine betaine in µmol/g fresh tissue, µg GB ml^−1^: glycine betaine reading obtained from standard curve in micrograms per ml, ml Dichloroethane: Dichloroethane vol used (9 ml), 102 µg µmol^−1^: Molecular weight of glycine betaine and: g samples, the weight of the plant sample used (0.5 g).

#### Measurement of malondialdehyde and hydrogen peroxide in leaves

To measure malondialdehyde, half a gram of fresh leaf sample was added into the falcon, then 5 ml of 50 mM potassium phosphate buffer (pH = 7) was added. Falcons were centrifuged at a speed of 14,000*g* at 4 °C for 30 min. Then 1 ml of supernatant was transferred to 2 ml tubes and 1 ml of 0.5% thiobarbituric acid solution containing 20% trichloroacetic acid was added. The mixture was then placed in a hot water bath at 65 °C for 30 min. At this stage, in order to stop the reaction, the tube was rapidly transferred in the ice bath for 30 min. The cooled mixture was centrifuged at 10,000*g* for 10 min at 4 °C. Finally, the absorbance of the mixture was measured by a plate reader at two wavelengths of 532 nm and 600 nm. The amount of malondialdehyde was expressed using the following equation [40].

$${\text{MDA}}\left( {{\text{nmol}}/{\text{g freash weight}}} \right) = \, [\left( {{\text{Abs 532 nm }}{-}{\text{ Abs 6}}00\;{\text{nm}}} \right)/\left( {{\text{QF }}*{\text{DF}}} \right)$$

MDA = Malondialdehyde content in nm/g fresh weight, QF = Quenching coefficient (155 mM/cm), DF = Dilution Factor (in this method is 20).

To measure hydrogen peroxide (H_2_O_2_), half a gram of fresh leaf in an ice bath was homogenized with 5 ml of 1% (w/v) trichloroacetic acid [20]. The homogenate mixture was centrifuged at 4 °C for 10 min at a speed of 10,000*g*. Then 1 ml of supernatant was added to half ml of 100 mM potassium phosphate buffer and 1 ml of 1 mM potassium iodide. Solution adsorption was measured at 390 nm [41].

### Statistical analysis of data

The irrigation and kaolin application experiment was performed as a factorial split based on randomized complete block design with three replications during 2017 and 2018. The main plot was deficit irrigation levels and sub-plot was kaolin concentrations. SAS statistical system software (ver. 9.4) was used to perform analysis of variance, and means were compared using Duncan’s test.

## Results

### Tree crown volume

**Fig. 1:**
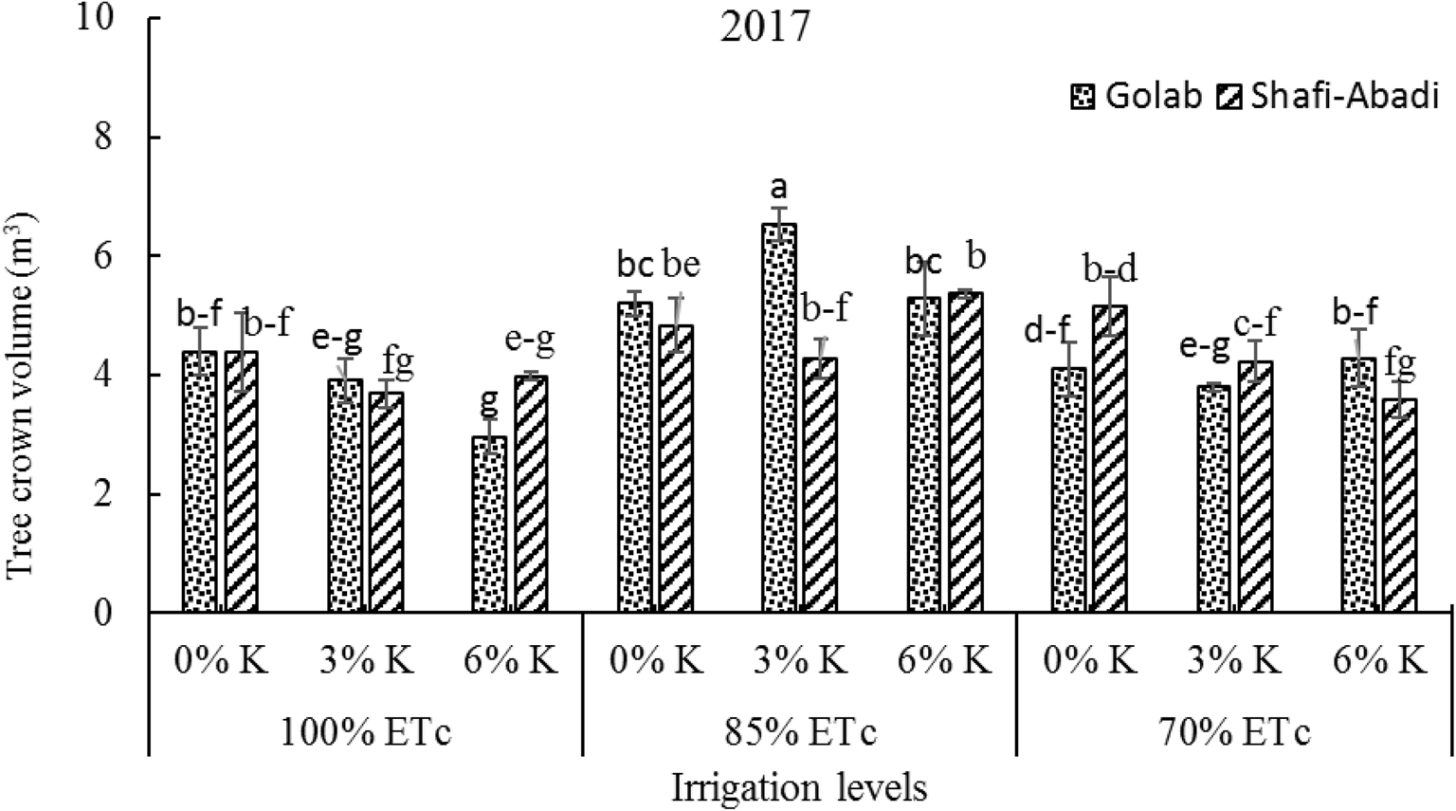
Effect of the irrigation levels (100%, 85% and 70% ETc) and kaolin application (0%, 3% and 6%) on tree crown volume of ʻGolabʼ and ʻShafi-Abadiʼ apples. Columns with the same letters are not significantly different at P < 0.05 level

The effect of irrigation and kaolin treatments on the crown volume of two apple cultivars in 2017 was investigated. Interaction of irrigation treatments × kaolin × cultivar had significant effect on tree crown volume (data not shown). Deficient irrigation at 85% ETc compared to 100% and 70% ETc treatments and 3% kaolin treatment at 85% ETc irrigation level improved apple tree crown volume in 2017 (Fig. 1). The lowest amount of tree crown volume was observed at 6% kaolin and 70% ETc treatment in apple cultivar Shafi Abadi at (Fig. 1).

### Physical properties of fruits

**Fig. 2:**
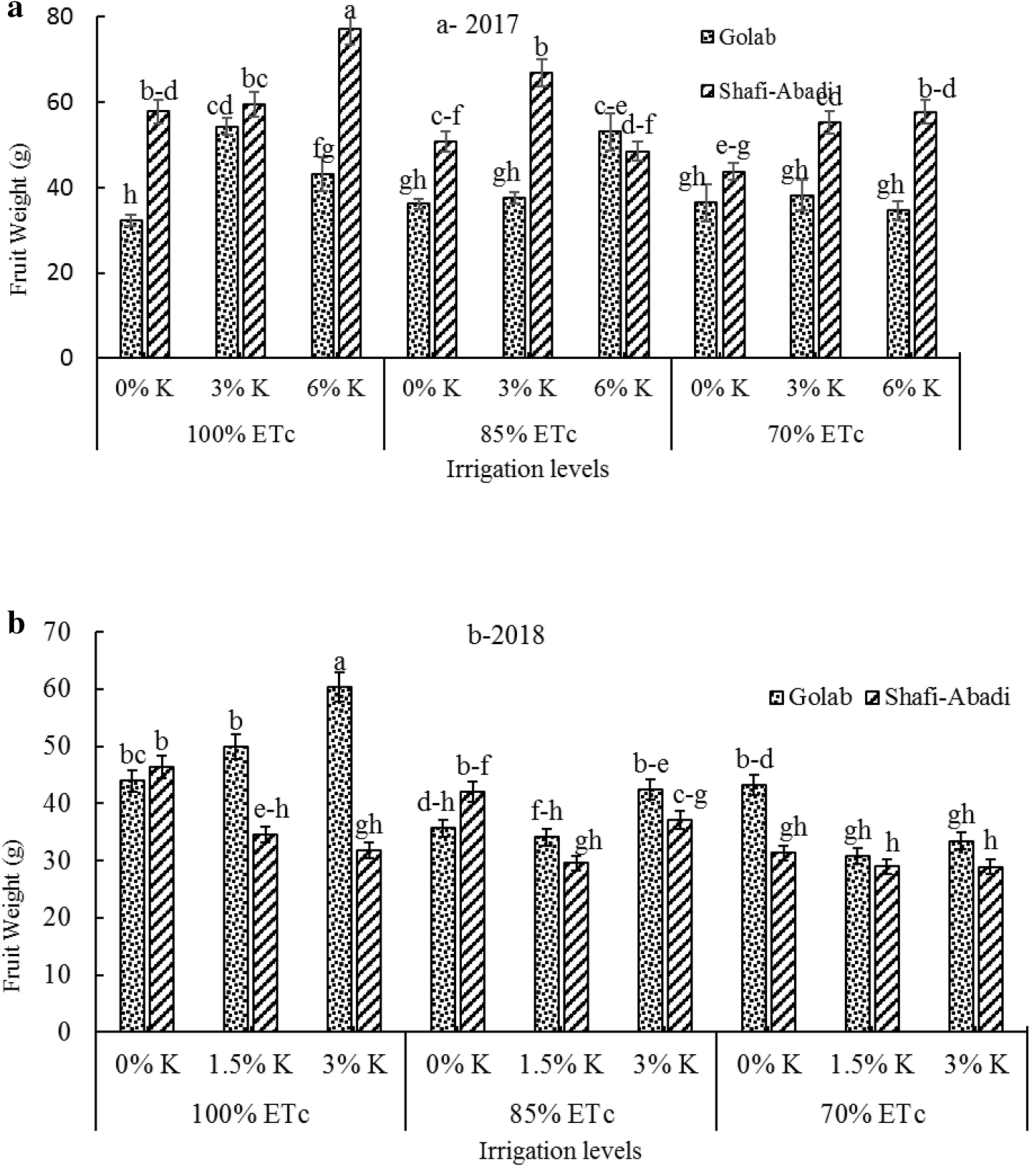
Effect of the irrigation levels (100%, 85% and 70% ETc) and kaolin application (0%, 3% and 6%) in 2017 (**a**) and (0%, 1.5% and 3%) in 2018 (**b**) on fruit weight of ʻGolabʼ and ʻShafi-Abadiʼ apples. Columns with the same letters are not significantly different at P < 0.05 level

Interaction effect of irrigation treatments × kaolin × cultivar in 2017 and 2018 on fruit weight was significant at 1% probability (data not shown). Deficit irrigation treatments significantly reduced fruit weight in both years (Fig. 2a and b). But kaolin treatments of 3 and 6 percent significantly increased fruit weight in 2017. In 2017, application of 6% kaolin increased Shafi Abadi fruit weight at 100% ETc irrigation (Fig. 2a). But in 2018, kaolin application of 3% at control irrigation significantly increased the apple fruit weight of Golab cultivar (Fig. 2b). At all irrigation levels, kaolin treatments increased apple fruit weight in 2017 but this increase was not consistent (Fig. 2a).

Comparison of fruit length showed that irrigation at 70% ETc decreased it in Shafi Abadi cultivar by 11% and 18% in 2017 and 2018, respectively, however 6% kaolin increased fruit length of both apple cultivars in 2017 (Table 1). The highest fruit length belonged to Shafi Abadi at full irrigation and 6% kaolin treatment with an average of 4.60 cm in 2017 (Table 1). Kaolin treatments of 1.5 and 3% at full irrigation level significantly increased fruit length of Golab cultivar in comparison to the control in 2018 (Table 1).

**Table 1:** Interaction of irrigation treatments (100%, 85% and 70% ETc), with kaolin application (0%, 3% and 6%) in 2017 and (0%, 1.5% and 3%) in 2018 and cultivar on some physical properties of ʻGolabʼand ʻShafi-Abadiʼ apples

Treatments	2017			2018	
	Fruit length (cm)		Fruit firmness (kg/cm^2^)	Fruit length (cm)	
	ʻGolabʼ	ʻShafi-Abadiʼ		ʻGolabʼ	ʻShafi-Abadiʼ
100% ETc					
0% Kaolin	3.50^d,e,f^ ± 0.03	4.27^a,b^ ± 0.01	6.27^bc^ ± 0.39	3.65^c,d,e^ ± 0.03	4.39^a^ ± 0.13
1.5% Kaolin	–	–	–	4.26^a,b^ ± 0.04	3.78^c,d,e^ ± 0.16
3% Kaolin	3.44^e,f^ ± 0.13	4.38^ab^ ± 0.11	6.81^b^ ± 0.31	4.27^a,b^ ± 0.23	3.59^c,d,e^ ± 0.15
6% Kaolin	3.46^d,e,f^ ± 0.04	4.60^a^ ± 0.16	5.91^c,d^ ± 0.23	–	–
85% ETc					
0% Kaolin	3.77^c,d,e^ ± 0.11	4.05^b,c^ ± 0.15	5.62^d^ ± 0.30	3.84^b,c,d,e^ ± 0.01	4.31^a,b^ ± 0.19
1.5% Kaolin	–	–	–	3.71^c,d,e^ ± 0.16	3.54^d,e^ ± 0.14
3% Kaolin	3.64^d,e^ ± 0.05	4.28^a,b^ ± 0.06	5.92^cd^ ± 0.20	3.96^a,b,c,d^ ± 0.07	3.78^c,d,e^ ± 0.03
6% Kaolin	4.21^b^ ± 0.17	4.07^b,c^ ± 0.02	6.70^b^ ± 0.62	–	–
70% ETc					
0% Kaolin	3.50^d,e,f^ ± 0.01	3.79^c,d^ ± 0.05	6.45^b,c^ ± 0.53	4.03^a,b,c^ ± 0.07	3.56^c,d,e^ ± 0.13
1.5% Kaolin	–	–	–	3.76^c,d,e^ ± 0.19	3.64^c,d,e^ ± 0.23
3% Kaolin	3.76^c,d,e^ ± 0.11	3.29^f^ ± 0.09	7.44^a^ ± 0.67	3.47^e^ ± 0.16	3.50^d,e^ ± 0.04
6% Kaolin	3.70^d,e^ ± 0.11	4.32^a,b^ ± 0.20	6.55^b^ ± _0.38_	–	–

	Fruit Firmness (kg/cm^2^)					
Treatments	100% ETc	85% ETc	70% ETc			
2017	6.54^a^ ± 0.36	5.77^b^ ± 0.25	6.94^a^ ± _0.63_			
2018	4.70^c^ ± 0.45	5.11^b,c^ ± 0.33	5.30^b,c^ ± 0.24			

Means within each column for each treatment followed by the same letters are not significantly different at *P *< 0.05

Firmness is one of the important qualitative characteristics of apple fruit that consumers pay close attention to when buying. It also increases the shelf life of the fruit. Severe deficit irrigation treatment (70% ETc) increased the firmness of apple fruit compared to the control and the mild deficit irrigation treatment, in the first year of experiment. In the first year, kaolin treatments also increased the firmness of fruit in this experiment. At all irrigation levels, 3% kaolin treatment increased apple fruit firmness in the first year of experiment (Table 1). There was no significant difference between irrigation treatments for the fruit firmness in the second year of experiment (Table 1).

Irrigation and kaolin treatments and their interactions in both 2017 and 2018 years had significant effects on infesttion of apple fruit with codling moth larva (data not shown). The results of this study showed that the most infected fruits in apple trees in 2017 were in 100% ETc treatment and in 2018 were in deficit irrigation treatments. In the first year study, 6% kaolin and in the second year 3% kaolin reduced 64.04% and 28.22% of infested fruits, respectively. In both year, ‘Shafi Abadi’ had fewer fruit infestations with worms than ‘Golab’ (Table 2).

**Table 2:** The effect of irrigation levels (100%, 85% and 70% ETc,) with kaolin application (0%, 3% and 6%) in 2017 and (0%, 1.5% and 3%) in 2018, and cultivar on fruit infestation with worm in ʻGolabʼand ʻShafi-Abadiʼ apples

Treatments	2017 Fruit infested with worms	2018 Fruit infested with worms
Irrigation treatments		
100% ETc	21.15^a^ ± 9.40	9.33^b^ ± 3.96
85% ETc	8.29^c^ ± 1.65	12.22^a^ ± 4.93
70% ETc	13.31^b^ ± 3.88	12.15^a^ ± 4.75
Kaolin		
0%	18.69^a^ ± 8.69	12.79^a^ ± 5.88
1.5%	–	11.75^a^ ± 4.06
3%	17.34^a^ ± 5.31	9.18^b^ ± 3.32
6%	6.72^b^ ± 1.41	–
Cultivar		
Golab	18.48^a^ ± 8.25	18.75^a^ ± 3.39
Shafi-Abadi	10.01^b^ ± 2.55	3.73^b^ ± 1.00

Means within each column for each treatment followed by the same letters are not significantly different at *P *≤ 0.05

The color index L* indicates brightness (or darkness) of color. Severe deficit irrigation treatment caused the apple fruit to shine in the first year but did not show significant effect in the second year. Kaolin treatment had no significant effect on fruit brightness in both years (Table 3). The 1.5% kaolin treatment at the full irrigation in 2018 had the highest apple fruit brightness (Table 3). Kaolin treatments increased the red index (a*) of apple fruit in the second year of experiment. Shafi Abadi cultivar had more red fruit index than Golab cultivar in both years (Table 3). Irrigation treatments, kaolin and cultivar, did not show significant effect on b* color index of these apple cultivars in both years of experiment (Table 3).

**Table 3:** The effect of irrigation treatments (100%, 85% and 70% ETc) with kaolin application (0%, 3% and 6%) in 2017 and (0%, 1.5% and 3%) in 2018, and cultivar on fruit skin colour of ʻGolabʼand ʻShafi-Abadiʼ apples

Treatments	2017			2018		
	Values L*	Values a*	Values b*	Values L*	Values a*	Values b*
Irrigation treatments						
100% ETc	69.98^a,b^ ± 1.43	− 10.5^a^ ± 2.40	37.51^a^ ± 0.93	69.83^a^ ± 1.75	− 8.97^a^ ± 4.29	40.73^a^ ± 1.92
85% ETc	68.92^b^ ± 1.16	− 10.54^a^ ± 3.15	37.28^a^ ± 1.06	69.43^a^ ± 1.67	− 8.40^a^ ± 3.73	40.00^a^ ± 1.90
70% ETc	70.17^a^ ± 1.76	− 9.94^a^ ± 1.81	38.33^a^ ± 1.17	69.89^a^ ± 0.83	− 7.30^a^ ± 4.44	41.79^a^ ± 1.60
Kaolin						
0%	69.89^a^ ± 1.48	− 9.86^a^ ± 2.40	37.91^a^ ± 1.25	69.35^a^ ± 1.36	− 10.06^b^ ± 2.92	41.05^a^ ± 2.27
1.5%	–	–	–	70.63^a^ ± 1.60	− 7.81^a^ ± 4.60	40.86^a^ ± 1.37
3%	69.69^a^ ± 1.53	− 10.51^a^ ± 2.55	37.85^a^ ± 1.01	69.18^a^ ± 1.35	− 6.79^a^ ± 4.61	40.60^a^ ± 1.79
6%	69.50^a^ ± 1.48	− 10.68^a^ ± 2.5	37.36^a^ ± 0.94	–	–	–
Cultivar						
Golab	71.69^a^ ± 1.04	− 13.70^b^ ± 1.43	37.60^a^ ± 0.89	69.64^a^ ± 1.22	− 15.41^b^ ± 1.10	39.95^b^ ± 0.95
Shafi-Abadi	67.70^b^ ± 1.12	− 7.00^a^ ± 2.14	37.81^a^ ± 1.22	69.79^a^ ± 1.68	− 1.03^a^ ± 2.49	41.72^a^ ± 2.32

Means within each column for each treatment followed by the same letters are not significantly different at *P *≤ 0.05

### Chemical properties of fruits

Interaction effect of irrigation treatment × kaolin × cultivar on fruit moisture content was significant at 1% probability level in 2017 (data not shown). Based on the comparison of means for fruit moisture content, ‘Shafi Abadi’ had the lowest value (41.88%) in the first year of experiment under severe deficit irrigation (70% ETc) and without kaolin (Table 4). Application of 3% and 6% kaolin on ‘Golab’ at 85% ETc irrigation level increased fruit moisture content 10.6% and 5.1% respectively in the first year experiment (Table 4).

**Table 4:** Interaction of irrigation treatments (100%, 85% and 70% ETc), kaolin application (0%, 3% and 6%) in 2017 and (0%, 1.5% and 3%) in 2018 and cultivar on some fruit biochemical characteristics of ʻGolabʼand ʻShafi-Abadiʼ apples

Treatments	2017				2018		
	Fruit moisture content		TA (g/100 ml)	TSS/TA	TSS	TA (g/100 ml)	TSS/TA
	ʻGolabʼ	ʻShafi-Abadiʼ					
100% ETc							
0% Kaolin	54.18^a^ ± 2.71	48.45^d,ef^ ± 0.27	0.14^c^ ± 0.02	8.43^a^ ± 1.26	8.00^c^ ± 0.39	0.11^c^ ± 0.01	6.39^c,d^ ± 0.49
1.5% Kaolin	–	–	–	–	10.93^b^ ± 0.60	0.20^a^ ± 0.04	7.01^b,c,d^ ± 1.88
3% Kaolin	54.07^a^ ± _0.96_	53.66^a,b^ ± 1.74	0.24^a^ ± 0.01	4.67^d^ ± 0.18	11.53^a,b^ ± 1.28	0.18^ab^ ± 0.03	7.35^a,b,c^ ± 2.04
6% Kaolin	53.21^a,b,c^ ± _0.91_	45.04^f,g,h^ ± 0.12	0.19^b^ ± 0.03	5.97^bc^ ± 1.04	–	–	–
85% ETc							
0% Kaolin	49.29^c,d,e^ ± _0.32_	49.73^b,c,d,e^ ± 0.86	0.19^b^ ± 0.02	5.80^bc^ ± 0.70	11.15^ab^ ± 0.70	0.18^b^ ± 0.03	6.23^d^ ± 1.15
1.5% Kaolin	–	–	–	–	11.35^a,b^ ± 0.77	0.17^b^ ± 0.03	7.37^a,b^ ± 1.52
3% Kaolin	54.52^a^ ± 1.09	46.96^e,f,g^ ± 0.90	0.20^b^ ± 0.02	5.71^bc^ ± 0.77	11.78^ab^ ± 0.77	0.18^b^ ± 0.02	6.86^b,c,d^ ± 1.17
6% Kaolin	51.83^a,b,c,d^ ± 1.23	49.16^c,d,e,f^ ± 1.27	0.20^b^ ± 0.02	5.49^c^ ± 0.70	–	–	–
70% ETc							
0% Kaolin	51.07^a,b,c,d,e^ ± 1.86	41.88 ^h^ ± 1.89	0.20^b^ ± 0.02	6.18^b^ ± 0.90	12.14^a^ ± 1.45	0.17^b^ ± 0.03	8.07^a^ ± 2.24
1.5% Kaolin	–	–	–	–	11.06^a,b^ ± 0.74	0.18^b^ ± 0.02	6.55^b,c,d^ ± 1.20
3% Kaolin	48.38^d,e,f^ ± _0.42_	43.73^g,h^ ± 1.24	0.20^b^ ± 0.02	6.34^b^ ± 0.76	10.85^b^ ± 0.78	0.18^b^ ± 0.02	6.39^c,d^ ± 1.07
6% Kaolin	49.75^b,c.d,e^ ± _0.71_	45.12^f,g,h^ ± 0.77	0.21^b^ ± 0.02	5.77^b,c^ ± 0.75	–	–	–

Treatments		2017					
		TSS					
	100% ETc	85% ETc	70% ETc	0% Kaolin		3% Kaolin	6% Kaolin
	11.15^b^ ± 0.37	11.00^b^ ± 0.36	11.96^a^ ± 0.42	11.32^a,b^ ± 0.51		11.61^a^ ± 0.34	11.17^b^ ± 0.43

The fruits under severe deficit irrigation treatment had more soluble solids (average of 11.96%) compared to control and the mild water deficit in the first year of experiment. But in the second year, both mild and severe irrigation treatments resulted to more soluble solids than control (Table 4). Kaolin treatments of 1.5% and 3% at full irrigation level significantly increased the soluble solids content of apples, but kaolin treatments under deficit irrigations did not show a significant effect on this trait in 2018 (Table 4).

Mild and severe irrigation restriction treatments significantly increased the titrable acid content of fruits in the first year but in the second year, they did not show significant effect on this trait (Table 4). The 3% and 6% kaolin treatments at full irrigation in the first year as well as the 1.5% and 3% kaolin treatments at full irrigation in the second year significantly increased the titrable acid content of the fruits (Table 4).

Severe water deficit treatment resulted to higher TSS/TA levels (as flavor index) than mild water deficit in the first year of experiment (Table 4). On the other hand, kaolin spraying in this experiment did not significantly affect the values of this index (Table 4). The lowest flavor index was observed in 3% kaolin treatment at full irrigation in 2017 and at 0% kaolin treatment at mild deficit irrigation in 2018 (Table 4).

### Relative water content

**Fig. 3:**
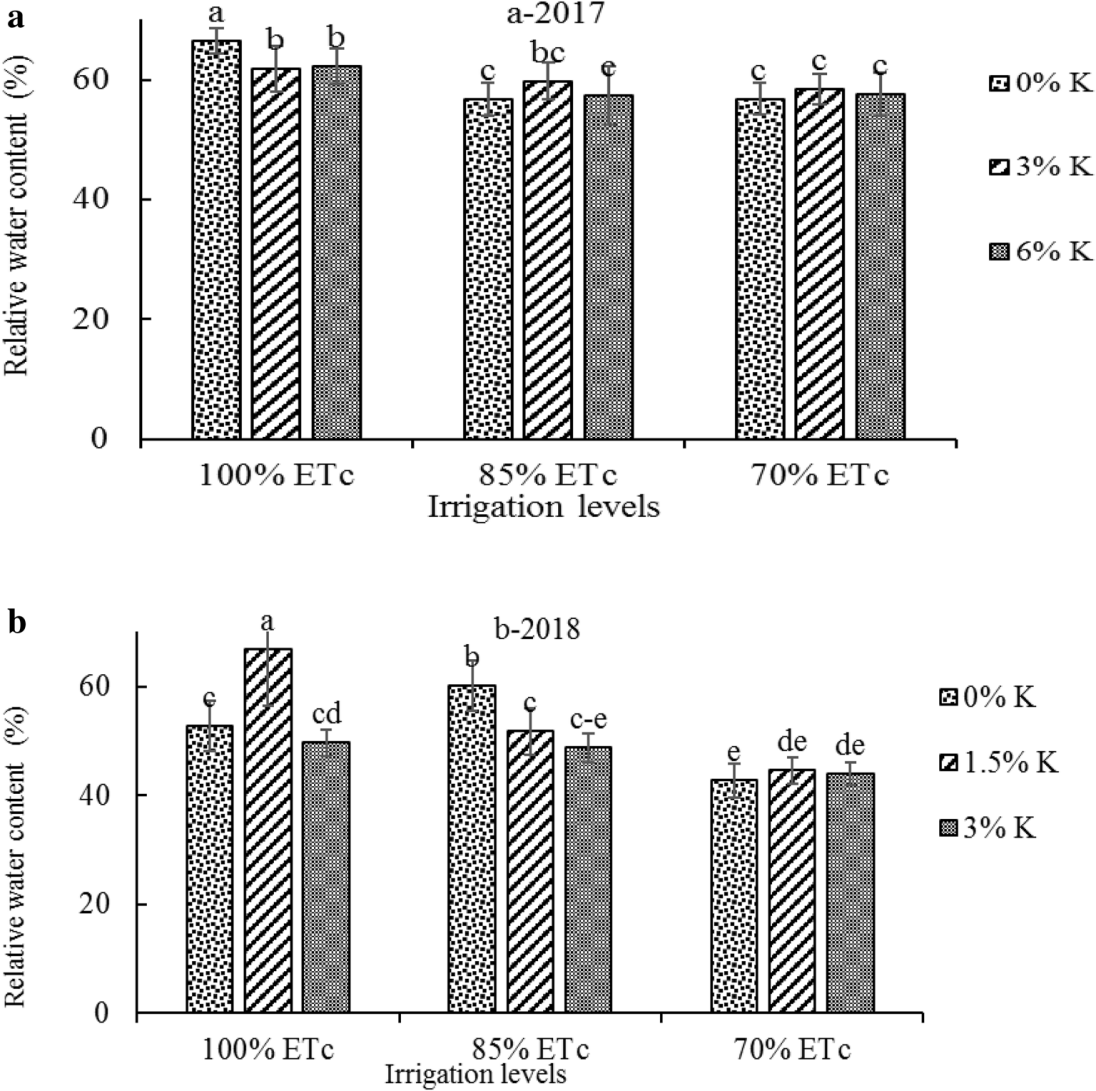
Interaction of irrigation treatments (100%, 85% and 70% ETc) and kaolin application (0%, 3% and 6%) in 2017 (**a**) and (0%, 1.5% and 3%) in 2018 (**b**) on leaves relative water content (RWC) of ʻGolabʼ and ʻShafi-Abadiʼ apples. Columns with the same letters are not significantly different at P < 0.05 level

Deficit irrigation treatments decreased leaf relative water content in both years (Fig. 3a and b). Kaolin treatments of 3 and 6% at full irrigation significantly reduced leaf relative water content in 2017 but at mild and severe irrigation, restrictions had no effect on this trait (Fig. 3a). But at the second year the 1.5% kaolin treatment increased the relative water content of leaf at 100% ETc irrigation (Fig. 3b).

### Proline and glycine betaine in leaves

Deficit irrigation treatments significantly increased leaf proline content compared to control in both years. Kaolin treatments increased leaf proline content in the first year but in the second year, their effect on leaf proline content was not significant. (Table 5). In the second year, irrigation treatments of 85% and 70% ETc without kaolin significantly increased leaf proline content compared to the other treatments (Table 5).

**Table 5:** Interaction of irrigation treatments (100%, 85% and 70% ETc), kaolin application (0%, 3% and 6%) in 2017 and (0%, 1.5% and 3%) in 2018 and cultivar on some biochemical characteristics of leaves of ʻGolabʼand ʻShafi-Abadiʼ apples

Treatments	2017				2018	
	Prolin (μmol g^−1^ FW	Glycine–betaine content (μmol g^−1^ FW)	MDA (nmol g^−1^ FW)	H_2_O_2_ content (μmol g^−1^ FW)	Prolin (μmol g^−1^ FW	MDA (nmol g^−1^ FW)
100% ETc						
0% Kaolin	4.09^d^ ± 0.41	24.01^bc^ ± 3.86	19.32^d^ ± 6.24	6.86^c,d^ ± 0.89	2.83^e^ ± 0.60	23.60^a^ ± 4.25
1.5% Kaolin	–	–	–	–	3.68^d,e^ ± 0.62	24.84^a^ ± 0.95
3% Kaolin	4.61^c,d^ ± 0.38	20.31^d^ ± 3.21	22.45^c^ ± 5.76	6.96^b,c,d^ ± 0.81	4.13^c,d^ ± 0.57	16.74^b^ ± 2.15
6% Kaolin	4.51^c,d^ ± 0.74	20.31^d^ ± 3.15	25.62^a,b^ ± 8.10	6.48^d^ ± 0.75	–	–
85% ETc						
0% Kaolin	4.72^c^ ± 0.49	22.97^c,d^ ± 3.05	23.08^b,c^ ± 4.71	7.64^a,b^ ± 1.60	5.13^c^ ± 0.44	25.00^a^ ± 3.88
1.5% Kaolin	–	–	–	–	4.99^c^ ± 0.46	17.73^b^ ± 3.23
3% Kaolin	5.00^b,c^ ± 0.45	24.76^b,c^ ± 3.89	24.05^bc^ ± 7.06	7.11^b,c,d^ ± 1.46	4.83^c,d^ ± 0.29	16.02^b^ ± 2.02
6% Kaolin	5.6^a^ ± 0.77	24.69^b,c^ ± 3.51	23.93^b,c^ ± 8.01	6.88^c,d^ ± 1.38	–	–
70% ETc						
0% Kaolin	5.35^a,b^ ± 0.62	23.38^b,c^ ± 4.14	27.14^a^ ± 5.22	7.32^b,c^ ± 1.35	8.05^a^ ± 0.22	23.73^a^ ± 5.68
1.5% Kaolin	–	–	–	–	6.69^b^ ± 0.64	16.33^b^ ± 1.08
3% Kaolin	5.39^a,b^ ± 0.61	25.94^b^ ± 3.74	28.34^a^ ± 5.51	7.64^ab^ ± 1.36	6.44^b^ ± 0.55	18.66^b^ ± 1.16
6% Kaolin	5.58^a,b^ ± 0.80	32.46^a^ ± 4.18	28.34^a^ ± 7.35	8.26^a^ ± 1.25	–	–

Means within each column for each treatment followed by the same letters are not significantly different at *P *≤ 0.05

Irrigation and kaolin treatments had significant effect on the glycine betaine content of leaf in the first year of experiment (Table 5). Irrigation treatment of 70% ETc and kaolin application of 3% and 6% resulted to the highest amount of glycine betaine of leaf in the first year of experiment (Table 5). The lowest glycine betaine content of leaf was observed in full irrigation plus 3% and 6% kaolin application (Table 5).

### Malondialdehyde and hydrogen peroxide in leaves

Malondialdehyde content increased in the first year with increasing deficit irrigation severity, but it did not show significant difference in the second year. Kaolin treatments of 1.5% and 3% in the second year significantly reduced the amount of malondialdehyde (Table 5). The highest content of malondialdehyde in the first year was at 70% ETc treatment combined with kaolin treatments of 3% and 6% and in the second year in non-kaolin treatments (Table 5).

The highest leaf hydrogen peroxide content was related to 70% ETc and 6% kaolin treatment with an average of 8.26 µmol g^− 1^ FW in 2017 (Table 5). The 6% kaolin treatment at 85% ETc deficit irrigation significantly reduced the hydrogen peroxide content of apple leaf compared to the 0% kaolin (Table 5).

## Discussion

Influence of kaolin and irrigation treatments on head volume of two apple cultivars (Golab and Shafi Abadi) was investigated in 2017. Severe drought stress led to decrease in growth of shoot length and diameter (data not shown), and 70% ETc treatment reduced vegetative growth compared to 85% ETc treatment (Fig. 1). The volume of apple cultivars had high correlation with shoot growth. Similar results have been reported by Dodd (2005) and was shown that stress conditions under deficit irrigation increased abscisic acid (ABA) biosynthesis in roots and decreased cytokinin synthesis in roots, shoots and buds [42]. There have been reports of decreasing vegetative growth in citrus trees [43], Japanese plum [44] and apricot [45] under water deficit conditions.

In the present study, kaolin treatments showed no significant effect on volume of apple trees (Fig. 1). However, some reports have shown that kaolin treatment enhances photosynthesis, accelerates carbon dioxide assimilation at noon, and increases vegetative growth due to improved light distribution within the canopy, reduced sun damage, and reduced shade temperature [46–48].

According to Fig. 2a and b, deficit irrigation treatments reduced fruit weight in both years. There have been many reports of apple fruit weight loss due to deficit irrigation applications [49–51], in olive [52] and in citrus [53]. Deficit irrigation decreases vegetative growth, photosynthesis rate and water and nutrient transfer to fruit, resulting in reduced fruit productivity and weight [54]. In the present study, kaolin treatments of 3% and 6% in the first year of experiment significantly increased fruit weight (Fig. 2a and b). As already reported, the fruits of ‘Empire’ apples treated with 3% and 6% kaolin were larger in size than the control [55]. Also, according to Wand et al. (2006), 3% kaolin treatment on apple trees reduced plant temperature and decreased water evaporation from fruit surface and consequently increased apple fruit size [27].

Fruit length in irrigation treatment of 70% ETc was reduced in Shafi Abadi cultivar in both years (Table 1). Similarly, sustained deficit irrigation during the growing season of pomegranate significantly reduced fruit size and decreased their economic value. However, sustained deficit irrigation was effective water saving strategy, but with negative effect of reduced fruit economic value due to their reduced size [2]. Similar results are also reported by application of deficit irrigation at the second stage of Japanese plum fruit growth [56]. Deficit irrigation treatments reduced apple fruit volume except when the trees were light crop [57]. In the present study, 6% kaolin treatment increased fruit length of both apple cultivars in 2017 (Table 1). Kaolin treated apples were reported to be larger in size [55]. The positive effect of kaolin on growth of pomegranate fruit was reported, but at high concentration of kaolin (5%) fruit size was not significantly different from control [29]. Kaolin appears to increase apple fruit size by reducing the amount of water evaporated from the fruit surface. It is likely that the condition created by kaolin causes less auxin degradation in the fruit and thus, resulted to more growth [27].

According to Table 1, severe deficit irrigation treatment (70% ETc) increased the firmness of apple fruit compared to control and the mild deficit irrigation treatment in the first year of experiment. It was previously reported that deficit irrigation treatments increased apple fruit firmness [58]. Water deficiency during the growth of citrus fruits resulted in the breakdown of cell wall polymers and further reduction of the osmotic potential of the fruit and ultimately the fruit firmness [53]. The results of the present study are in line with the findings of researchers who considered that kaolin application on apple trees is a factor in contributing to a slight increase in fruit firmness [27, 59].

According to Table 2, the lowest fruit infestation with codling moth in 2017 was related to deficit irrigation treatments (85 and 70% ETc). Similarly deficit irrigation treatments have been reported to reduce grape fruit pests [60]. However, in the second year (2018) the infestation was increased (Table 3). The reason could be the weakness of trees and the high temperature stresses in the second year. Kaolin treatment of 6% in the first year and kaolin treatment of 3% in the second year also reduced (6.27% and 9.18%) the infestation of fruits in this study (Table 2). The kaolin particles on fruit cling to the insect’s foot, disrupting their movement feeding and egg laying. Also, kaolin has a physiological repellency due to its white color and light reflection that reduces the attractiveness of the tree and fruits to the pests [61]. The use of kaolin, at a concentration of 3% on pear trees reduced the population of pear psylla by 59% to 82% [62]. Also, the amount of pomegranate infestation with *Ectomyelois ceratoniae* decreased by kaolin application [63]. According to a report, kaolin application reduced apple powdery mildew but increased woolly apple aphid compared to control [31].

Severe deficit irrigation treatment increased the color index a* in the second year of experiment (Table 3). Deficit irrigation treatment has been reported to improve the fruit color of apples. The red color of apple is due to the presence of anthocyanins, which are stimulated by light and cool temperatures. Severe deficit irrigation controls vegetative growth and reduces competition for photosynthetic products between fruits and vegetative organs, as well as the better penetration of light into the crown. These result to higher fruit sugar accumulation, which plays an important role in the anthocyanin formation [5, 7, 12, 58]. Deficit irrigation on peach and apricot fruit growth stages resulted to a more intense redness of the fruit skin and earlier fruit ripening [4, 64]. Kaolin treatments also increased the redness index of apple fruit (a*) in the second year of experiment (Table 3). It has been reported that kaolin reduces nighttime temperature and respiration, which increases the accumulation of sugar in the fruit to produce anthocyanin [23]. Researchers believe that the main effect of temperature on anthocyanin formation is affecting the activity of PAL (Phenylalanine ammonia-lyase), such that lower temperatures increase its activity [24, 65].

According to the fruit moisture content percentage, ‘Shafi Abadi’ had the lowest value in the first year of experiment under severe deficit irrigation (70% ETc) and without kaolin (Table 4). Severe drought stress may be the reason for the decrease in fruit moisture content during the first year of experiment. Contrary to the present results, it was reported that moisture content of pomegranate fruit arils under sustained and regulated deficit irrigation did not significantly differ from control [66]. Plant structure and genetics, climatic conditions as well as soil conditions appear to influence this feature. Foliar application of 3% and 6% kaolin on Golab cultivar at 100% ETc irrigation showed no significant difference with control on fruit moisture content (Table 4). Similarly, it is reported that there were no significant differences between pomegranate trees sprayed with kaolin and control in terms of qualitative factors such as fruit weight, fruit moisture content and fruit dry weight [29].

In present study, the fruits of severe water deficit treatment showed 7.2% increase in soluble solids compared to control in the first year of experiment (Table 4). Conversion of starch to simpler sugars and their accumulation due to water deficit stress could be the reason for increasing total soluble solids (TSS). Also, less vegetative growth under water deficit, may result to more accumulation in fruit TSS. In cherry fruit [35] reduced irrigation (up to 30% of water requirement) from the time of pit hardening to harvesting increased the quality of fruit including soluble solids [67]. In citrus fruits, as the amount of irrigation increased, fruit size and weight increased, but soluble solids and acids decreased [68].

Kaolin application of 1.5% and 3% at full irrigation resulted to increased soluble solids content of apple fruit in 2018 by 36.6% and 44.1%, respectively (Table 4). It is mentioned that foliar application of kaolin reduced the leaf and fruit temperature of apples and decreased respiration rate, resulting to less carbohydrate degradation and the possibility of its more allocation to fruits [69]. According to other reports, kaolin application on ‘Crisp Pink’ apple had no significant effect on TSS [59] whereas in ‘Anna’ apple TSS decreased with increasing kaolin concentration (0, 2, and 3%), but it did not affect total acids (TA) [70]. The decrease in TSS due to the application of kaolin is attributed to increased fruit size, delayed maturation and ripening of the fruit, delayed conversion of starch to sugar, and decreased assimilation [59, 70]. Deficit irrigation treatments significantly increased the titrable acids content of fruits in the first year but in the second year did not show any significant effect on this trait (Table 4). The effect of water deficit on TA values is very vague and unclear. In some reports, increase in TA under deficit irrigation has been reported, as in grape [71]. Kaolin treatments in both years under full irrigation significantly increased the titrable acid content of fruits (Table 4). In strawberries, deficit irrigation treatments increased total sugar concentration and some fruit acids [72]. Apple fruit acidity is mainly due to the malic acid, and an increase in temperature reduces its content, hence, a decrease in temperature by kaolin application can prevent the reduction of apple acids [73].

Deficit irrigation treatments reduced relative water content (RWC) of leaf in both years (Fig. 3a and b), which is due to the lower water absorption. Reduced leaf RWC in apple tress under water deficit has been reported [51]. Decrease in RWC of olive leaves with increasing the period of drought stress was about 50% at the end of the drought stress period compared to the control plants [74]. Reduction in leaves RWC, may led to decreased stomatal conductance and carbon dioxide entry into the leaf mesophyll, ultimately less plant photosynthetic efficiency [75, 76].

According to Table 5, deficit irrigation treatments significantly increased leaf proline content in both years. Proline participates in the stabilization of subcellular structures (such as membranes and proteins) and the control of free radicals under stress conditions. Proline rapidly regulates the changes in the cell’s aquatic environment and prevents leaf water loss [77, 78]. Water deficit stress on apples showed that it had a significant effect on morphological, physiological and biochemical characteristics of trees. Sever deficit irrigation increased catalase activity, anthocyanin and proline content of apple leaves [51]. Kaolin treatment in the first year, increased leaf proline, while in the second year, leaf proline was not significant (Table 5). Irrigation treatments of 85% and 70% ETc without kaolin application significantly increased leaf proline compared to the other treatments in the second year. Contrary to the results of present study, application of 3% and 6% kaolin on walnut trees reduced leaf proline [30] which might be due to differences in plants, stress severity and environmental conditions. Also in grape, 5% kaolin significantly reduced proline content of leaves compared to control [79].

Results of this study showed that the amount of glycine betaine increased due to deficit water stress. In the first year of experiment, deficit irrigation treatment of 70% ETc with both kaolin concentrations of 3% and 6% resulted to the highest amount of glycine betaine in apple leaves (Table 5). The lowest leaf glycine betaine was observed in 100% ETc treatment with 3 and 6% kaolin (Table 5). Glycine betaine maintains cell membrane integrity under stress conditions through its role in reducing the production of reactive oxygen species. The production of compounds such as proline and glycine betaine maintain osmotic balance in the cell [80, 81]. Similar response to drought stress by accumulating compatible solutes including glycine betaine, which modify osmotic pressure is reported in most plants, as well as perennials [82]. The negative effects of drought stress in olive trees sprayed with kaolin and glycine betaine were reduced [83].

In the present study, malondialdehyde content increased in the first year with increasing deficit irrigation level but did not show significant effect in the second year (Table 5). Increased malondialdehyde is a good indicator of the severity of oxidative damage to fatty acids of membranes. With the increased oxidation of lipids, damage to membrane and consequently electrolyte leakage is increased [84]. Kaolin treatments significantly reduced malondialdehyde levels in the second year (Table 5). As reported, by activating enzymatic systems in plants, kaolin protects the plant against environmental stresses and the cell membrane [85]. Wheat plant under drought stress treated with 3% kaolin foliar application had significantly lower levels of malondialdehyde than wheat without kaolin. In grape also 5% kaolin treatment significantly decreased lipid peroxidation of leaves compared to control [79].

The highest amount of leaf hydrogen peroxide was related to 70% ETc and 6% kaolin in 2017 with an average of 8.26 μmol g^−1^ FW (Table 5). Kaolin application of 6% under 85% ETc irrigation significantly reduced the amount of hydrogen peroxide in apple leaf compared to 0% kaolin (Table 5). Hydrogen peroxide damages biological molecules such as lipids, proteins, and nucleic acids. Drought stress significantly increased the amount of hydrogen peroxide in the apple leaves, leading to increasing the activity of antioxidant enzymes such as superoxide dismutase, catalase, ascorbate peroxidase and glutathione reductase [78]. Also in grape, application of kaolin increased the activity of antioxidant enzymes and defense system under summer heat stress and resulted in a significant decrease in the activity of reactive oxygen species including hydrogen peroxide in leaves [79].

## Conclusion

Using a sustained deficit irrigation strategy (mild drought throughout the growing season) will save water. The results of this study showed that irrigation application at 85% ETc compared to 100% and 70% ETc plus 3% kaolin application will improve many features of apple leaves and fruits.

## Data Availability

Not applicable.

## Abbreviations

DI: Deficit irrigation
SDI: Sustained deficit irrigation
